# Measuring synchronous bursting and spiking under varying second order network connectivity statistics

**DOI:** 10.1186/1471-2202-15-S1-P56

**Published:** 2014-07-21

**Authors:** David Burstein, Jonathan Rubin

**Affiliations:** 1 Department of Mathematics, University of Pittsburgh, Pittsburgh, PA 15217, USA

## 

Synchronous bursting plays an integral role in a variety of applications, from generating respiratory rhythms and inducing hormonal releases to conveying information about a stimulus. Since the network structure influences the dynamical behavior of a network, we aim to identify network structures that promote synchronous bursting.

By generating the network topology using specific probability distributions, Zhao et al. [1] demonstrated that the prevalence of certain two-connection network motifs significantly affects synchronous spiking. We build on these results by constructing a new graph theoretical method for quantifying how the network topology promotes synchronous spiking and bursting. We first verify the validity of the new measure by establishing consistency with the results of Zhao et al. By utilizing this new measure, our gains are two-fold. This new measure not only generalizes prior results on synchronous spiking, as the framework can accommodate network topologies generated by any probability distribution, but also provides a deterministic method for quantifying the likelihood of synchronous bursting under various network topologies.

We construct the graph theoretical measure by restricting the numbers of incoming and outgoing connections of a neuron to take one of finitely many possible values. Fixing certain constraints, such as the number of neurons in our network and the expected number of connections, we construct a probability distribution for our network. Through averaging, we construct difference equations from the probability distribution to track the expected number of active neurons and inactive neurons in a given time step. In the special case of spiking, the difference equations force a neuron to fire if one of its neighbors fires. By considering the maximum number of active neurons within a single time step, we formulate a graph theoretical measure for synchronous spiking. Using an analogous system of difference equations, we can construct a graph theoretical measure for synchronous bursting as well. Consequently, by calculating the second order network statistics from the probability distribution, and identifying all candidate probability distributions that satisfy the constrains, we can analyze how second order network statistics from any probability distribution promote synchronous spiking and bursting.

In our simulations, we find that increasing the covariance of the in-degree and out-degree monotonically increases the predicted occurrence of synchronous spiking under the graph theoretic measure. We also note that for a wide range of parameters, the number of neurons in the network and the constraints on the governing probability distribution of our network have minimal qualitative impact on the relationship between second order network statistics and predicted likelihood of synchronous spiking. Furthermore, preliminary results regarding the effect of second order network statistics on predicted synchronous bursting suggest a more intricate relationship than in the case of synchronous spiking. Based on the consistency of the measure in the case of synchronous spiking with the existing literature and due to its ability to incorporate relatively abstract properties of the network, we conjecture that the measure will provide new insight regarding the impact of the network topology on continuous time-scale models of synchronous bursting and other complex behaviors.
